# Unexpected spatial impact of treatment plant discharges induced by episodic hydrodynamic events: Modelling Lagrangian transport of fine particles by Northern Current intrusions in the bays of Marseille (France)

**DOI:** 10.1371/journal.pone.0195257

**Published:** 2018-04-25

**Authors:** Bertrand Millet, Christel Pinazo, Daniela Banaru, Rémi Pagès, Pierre Guiart, Ivane Pairaud

**Affiliations:** 1 Mediterranean Institute of Oceanography UM 110, Aix-Marseille Université, CNRS, IRD, Campus de Luminy, Marseille, France; 2 Mediterranean Institute of Oceanography UM 110, Université de Toulon, CNRS, IRD, La Garde, France; 3 Laboratoire Environnement Ressources Provence Azur Corse, Institut Français de Recherche pour l′Exploitation de la Mer, La Seyne-sur-Mer, France; 4 Laboratoire DYNECO/DHYSED, Institut Français de Recherche pour l′Exploitation de la Mer, Plouzané, France; Universidade de Aveiro, PORTUGAL

## Abstract

Our study highlights the Lagrangian transport of solid particles discharged at the Marseille Wastewater Treatment Plant (WWTP), located at Cortiou on the southern coastline. We focused on episodic situations characterized by a coastal circulation pattern induced by intrusion events of the Northern Current (NC) on the continental shelf, associated with SE wind regimes. We computed, using MARS3D-RHOMA and ICHTHYOP models, the particle trajectories from a patch of 5.10^4^ passive and conservative fine particles released at the WWTP outlet, during 2 chosen representative periods of intrusion of the NC in June 2008 and in October 2011, associated with S-SE and E-SE winds, respectively. Unexpected results highlighted that the amount of particles reaching the vulnerable shorelines of both northern and southern bays accounted for 21.2% and 46.3% of the WWTP initial patch, in June 2008 and October 2011, respectively. Finally, a conceptual diagram is proposed to highlight the mechanisms of dispersion within the bays of Marseille of the fine particles released at the WWTP outlet that have long been underestimated.

## Introduction

Large coastal cities are a significant source of marine pollution in the Mediterranean Sea. Metal and organic pollutants are discharged into the marine environment through the sewage system and from the surrounding catchments. They are released as diffusive inputs or during flood events. The city of Marseille has one of the largest Wastewater Treatment Plants (WWTP) in Europe, serving 1.7 million inhabitants and using both physical and biological treatment processes. A large proportion of the treated wastewaters are discharged into the rivers (50%), with a noticeable signature of trace metals from the WWTP effluent during baseflow [1]. In dry periods, continental outflows from urban and industrial areas merge in Marseille and mix with the WWTP effluent down to an outlet located at the sea surface at Cortiou on the southern coastline. During flood events, significant amounts of trace metals are discharged into the surface aquatic system through runoff processes [2,3], and in extreme cases as the outflows exceed the WWTP outlet capacity, a significant part of the continental waters are directly channeled into the southern bay of Marseille. On average, during flood events, 90% of the continental waters and particulate matter are channeled through the outlet and only 10% are diverted to the southern bay. In addition, the discharge of untreated wastewater in the coastal zone was estimated between 456 and 1450 t^**.**^y^-1^ of Suspended Particulate Matter during the period 2001–2007 [4]. As the WWTP effluent is a major source of water and particles discharged from the city to the coastal area, previous modelling work focused on Marseille as a source of nutrients or contaminants for the coastal area. The focus was on modelling the impact of meteorological and hydrodynamic conditions on the fate of the WWTP contaminants in the coastal waters [5,3].

Successive modelling studies of the hydrodynamics in the bays of Marseille have been carried out. Firstly, we used the 3D-Princeton Ocean Model on a 250 x 350 horizontal grid with a horizontal resolution of 100 m and 11 vertical sigma levels, in order to map the wind**-**induced hydrodynamic connections between the artificial reefs submerged in the central southern bay and the surrounding shorelines [6,7]. Secondly, 2 versions of the 3D-model MARS3D–RHOMA (for Rhône-Marseille Area), with respective resolutions of 200 m and 400 m and 30 vertical sigma levels, were implemented on a domain extending from the Rhône River (roughly 40 km west of Marseille) to Cap Sicié (roughly 40 km southeast of Marseille), within the framework of the METROC, FP7 PERSEUS and EC2CO/PNEC MASSILIA projects, with the aim of coupling hydrodynamic, sediment transport and biogeochemical models. The results, described in [5,8**–**11], confirmed that the water circulation in the bays of Marseille is mainly controlled by the wind regimes, but also by the Rhône River seasonal floods, and the periodic intrusions of the Northern Current (NC) on the continental shelf south of Marseille. These intrusions of the NC on the continental shelf have been studied and explained by many authors on the basis of surveys involving in situ Acoustic Doppler Current Profiler (ADCP) measurements and 3D numerical modelling [12**–**15]. The latter results led the authors [15] to propose 3 types of wind conditions likely to favor an intrusion of the NC on the shelf: i) ‘intrusions under easterlies’ that correspond to a stable SE wind regime; ii) ‘intrusions under northwesterlies’ that correspond to a relaxation of a NW wind, in stratified conditions; iii) ‘a combination of the 2 wind patterns’ involving a strong NW wind event immediately followed by a SE wind regime that reinforces the intrusion of the NC on the shelf.

In addition, a recent combination of the results of the 3D RHOMA model (resolution of 200 m) and the Lagrangian model ICHTHYOP was used to compute the wind**-**induced connectivity at the surface between different populations of the seaweed *Cystoseira amentacea* within the bays of Marseille under strong and well-established NW and SE wind regimes [16]. The results highlighted very rapid northwestward connections of propagules, considered as passive particles, at the surface in less than 18 hours across the bays from the WWTP outlet at Cortiou to the limit of the northern coastline. However, this modeling experiment showed that particle trajectories induced by well-established SE winds gave rise to strong connections between the eastern and western limits of the domain, passing south of the Frioul archipelago, but without entering the inner bays of Marseille and not resulting in connections on the shorelines of the northern and southern bays [16].

As a result, more recent simulations of connectivity were performed by combining the results of the RHOMA model (200 m resolution) with the ICHTHYOP tool, still under efficient SE wind regimes, but this time taking into account the deeper sub**-**surface circulation between 10 and 30 m depth, likely to enter the inner bays. These new sets of computations of the sub**-**surface circulation entering the inner bays of Marseille provided a basis for only taking into account the episodes of intrusions of the NC on the continental shelf that occurred under SE wind regimes, which have emerged as the dominant process giving rise to connections between southeastern offshore areas and the northern and southern bays. This result is of major importance in that it concerns the inner bays of Marseille, characterized by the highest number of sites that are vulnerable with regard to human activities such as tourism and the commercial activities of ports or beaches, fish farming, artificial reefs for production or sampling stations for water quality monitoring.

The aim of this study was to provide new insight into the previously under-estimated impact of the Cortiou WWTP effluent on the water quality of the bays of Marseille. Our approach was not chosen according to the usual approach defined in terms of the dilution of dissolved constituents, but according to a Lagrangian approach that consisted in computing the trajectories of a patch of passive and conservative particles discharged at the WWTP outlet, under the specific conditions of circulation which characterize the intrusion of the NC on the continental shelf near Marseille. The impact of the WWTP effluent on the water quality of the bays is identified, both from the spatial and quantitative points of view, by quantifying the amounts of fine, non-motile, neutrally-buoyant particles that reach 10 different specific sites, chosen along the shorelines of both northern and southern bays, according to their vulnerability with regard to local human activities.

## Materials and methods

### Study area

**Fig 1:**
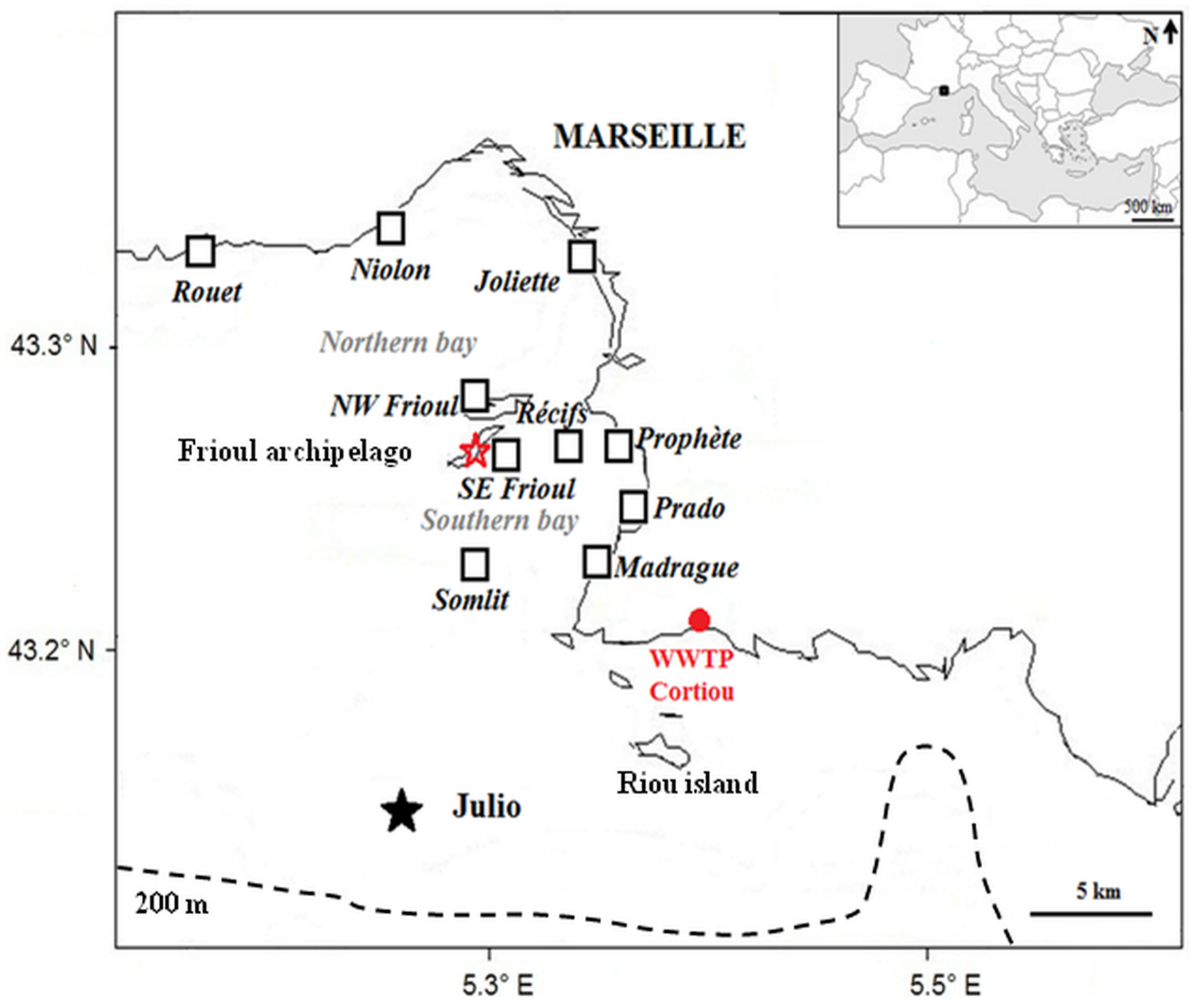
General map of the study area. Black squares indicate the 10 chosen vulnerable areas where particles transported from the WWTP were counted during the Lagrangian computations; *red star* indicates the Frioul meteorological station; *black star* indicates the Julio station (bottom-moored ADCP); *red point* indicates the WWTP outlet; *dotted black line* refers to the 200 m isobath.

Fig 1 presents the spatial range of our study site, limited to the northern and southern bays of Marseille, including the central Frioul archipelago, where a meteorological station is located, and the WWTP outlet located at Cortiou (43.213°N; 5.40318°E). Fig 1 also shows the site ‘Julio’,100 m isobath at 43.135°N; 5.255°E [17], where a bottom-moored ADCP measured the currents associated with intrusions of the NC on the continental shelf between February 2012, and April 2015 [12,15].

In addition, Fig 1 presents the locations of the 10 similar square boxes of 650 m (4.2x10^5^ m^2^), which were chosen all along the shoreline of both the northern and southern bays as representative of the most vulnerable areas with regard to human activities likely to be affected by particles transported from the WWTP outlet. Table 1 presents the coordinates of the 10 boxes considered, which represent harbours (Madrague, Niolon, Joliette); beaches (Rouet, NW Frioul, Prophète, Prado), fish farming (SE Frioul), artificial reefs (Récifs) and a sampling station for water quality monitoring (Somlit).

**Table 1:** Coordinates of the 10 vulnerable areas where particles arriving are counted.

locations and human activities	NW corner coordinates	SE corner coordinates
1- Somlit (sampling station)	*43*.*240°N / 5*.*292°E*	*43*.*234°N / 5*.*300°E*
2- SE Frioul (fish farming)	*43*.*272°N / 5*.*303°E*	*43*.*266°N / 5*.*311°E*
3- Madrague (harbour *and bathing*)	*43*.*241°N / 5*.*352°E*	*43*.*235°N / 5*.*360°E*
4- Niolon (harbour *and bathing*)	*43*.*338°N / 5*.*251°E*	*43*.*332°N / 5*.*259°E*
5- NW Frioul (bathing)	*43*.*289°N / 5*.*295°E*	*43*.*283°N / 5*.*303°E*
6- Récifs (artificial reefs)	*43*.*277°N / 5*.*336°E*	*43*.*271°N / 5*.*344°E*
7- Prophète (bathing)	*43*.*274°N / 5*.*359°E*	*43*.*268°N / 5*.*367°E*
8- Prado (bathing)	*43*.*258°N / 5*.*367°E*	*43*.*252°N / 5*.*375°E*
9- Rouet (bathing)	*43*.*335°N / 5*.*173°E*	*43*.*329°N / 5*.*181°E*
10- Joliette (harbour)	*43*.*317°N / 5*.*352°E*	*43*.*311°N / 5*.*360°E*

### Winds

**Fig 2:**
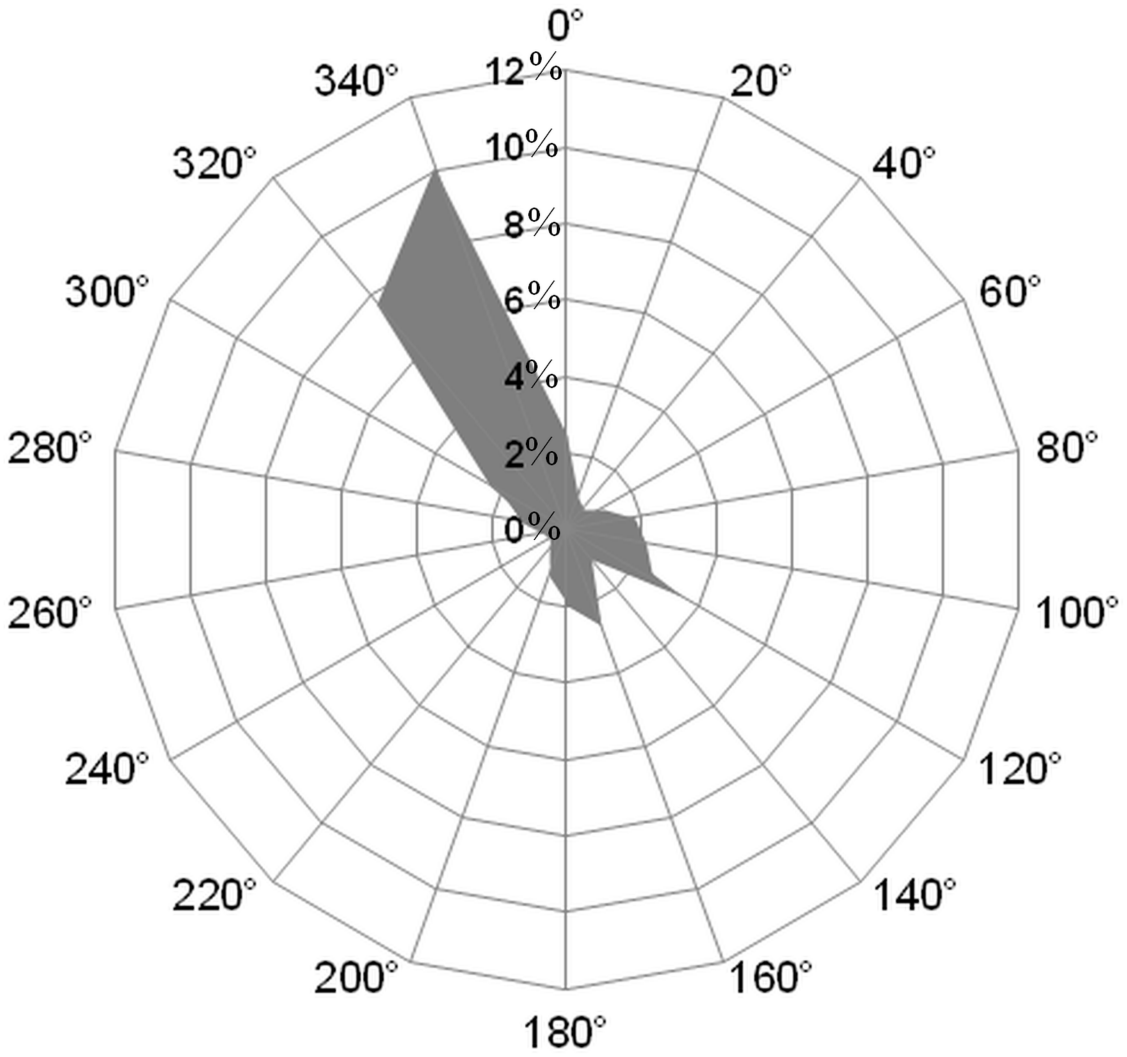
Wind rose measured at the Frioul meteorological station (altitude: 74 m). Frequency distribution of directions from which hourly averaged winds with speeds > 2 m^**.**^s^-1^ originated during 1976–1998, measured clockwise from north in degrees.

The local wind regime was observed at the Frioul meteorological station, located in the Frioul archipelago at the center of the domain, 74 m above sea level (Fig 1) and managed by the Pytheas Institute of Aix-Marseille University. Fig 2 presents the corresponding wind rose that highlights the 2 prevailing NW 300°-340° and SE 100°-160° wind sectors, with frequencies of occurrence in time over the 1976–1998 period for hourly averaged wind speeds greater than 2 m^**.**^s^-1^. The frequencies of occurrence are 38.1% and 21.8% for the NW 300°-340° and SE 100°-160° sectors, respectively.

**Fig 3:**
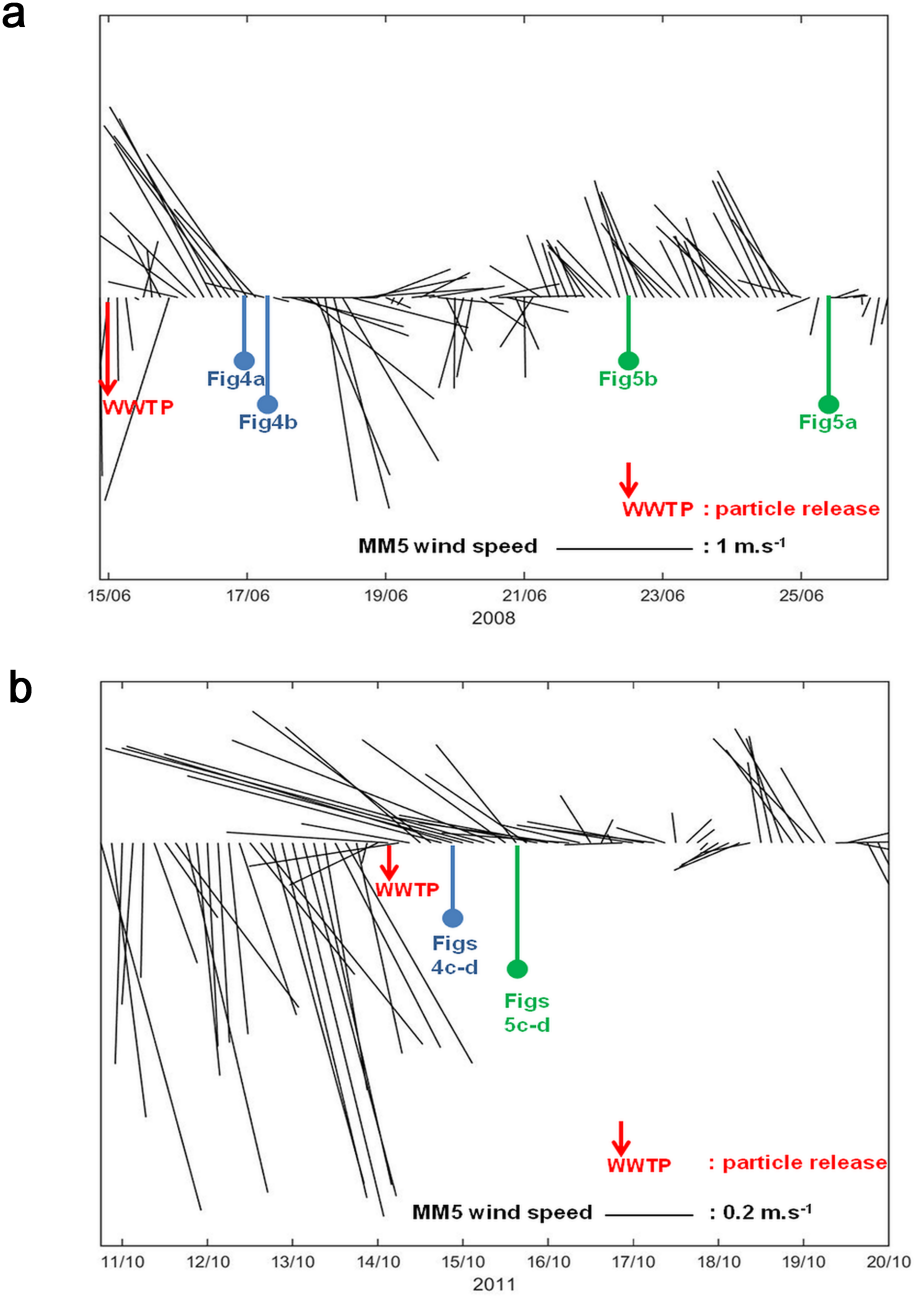
Computed MM5 winds (10 m above sea level) at Somlit station. MM5 wind intensities and directions, clockwise from the north in degrees, computed (3 km resolution) every 3 hours at 10 m above sea level at a location corresponding to Somlit station; a) forcing conditions for the computation of June 2008; b) forcing conditions for the computation of October 2011; times for particle release (WWTP outlet) and model outputs (current fields and particle trajectories) are mentioned in each time series; Note the different scale of wind vectors in Figs 3a and 3b.

In addition, each period of computation of water circulation and Lagrangian transport was chosen in relation to the wind forcing conditions of our model. Fig 3 presents the time series during the 2 periods chosen in our study, at a location corresponding to Somlit station (Fig 1), of the wind conditions computed every 3 hours at 10 m above sea level by the MM5 meteorological model (3 km resolution wind conditions of the RHOMA model). During the period of June 2008 (Fig 3a), the wind conditions were characterized by a 10-day period of moderate S-SE 130°-170° winds, corresponding to hourly averaged speeds < 7 m.s^-1^ measured at the Frioul meteorological station 74 m above sea level, from about 2 days before the particle release until the end of the particle dispersion, from June 15 2008 to June 25 2008. This period is interspersed with periods of unstable and weak NW winds, from June 18 2008 to June 21 2008. During the period of October 2011 (Fig 3b), the wind conditions were characterized by a strong NW winds event, from October 7 2011 to October 14 2011, followed by weak E-SE winds as reported in [11] and corresponding to hourly averaged speeds < 5 m.s^-1^ measured at the Frioul meteorological station (74 m above sea level). After the particle release on October 14 2011, the wind conditions presented weak and unstable E-SE 80°-110° winds until the end of the particle dispersion on October 19 2011.

### NC Intrusions

It should be noted that our study does not claim to be representative for all intrusion events, which would require intensive statistical work, and would offer conclusions which would still involve uncertainties as the definition of an “intrusion” is not easy to match with realistic situations. Only typical situations are considered here, with the associated limitations.

On the one hand, according to [12] and from an academic point of view, an intrusion of the NC on the continental shelf south of Marseille is defined as a branch of the NC crossing onshore (northwestwards) the 200 m isobath between 5.1° E and 5.8° E (Fig 1). We took into account this criterion to choose our study periods on the basis of the current fields calculated by the RHOMA model (see below).

On the other hand, according to [15], among the three types of wind conditions liable to favor an intrusion of the NC on the continental shelf, only the 2 situations mentioned as i) ‘intrusions under easterlies’ and iii) ‘a combination of the 2 wind patterns’, involve the forcing of a SE wind regime, and are the only situations that allow particles to be transported from the WWTP effluent to the inner bays of Marseille.

Therefore, for our study focused on the particle connections within the bays, we selected 2 periods characterized by 2 different SE wind conditions representative of the local variability (Fig 2): firstly, a period of 22 days from June 15, 0:00, to July 7, 0:00, 2008 and secondly, a period of 5 days and 18 hours from October 14, 6:00, to October 20, 0:00, 2011. These periods are characterized by intense intrusions of the NC on the continental shelf, and specific SE wind regimes: the period of June 2008 is characterized by 10 days of quite stable S-SE winds interspersed with periods of unstable and weak NW winds (Fig 3a), which is representative of the type i) intrusion, according to [15]; the second period in October 2011 is characterized by 5 days of E-SE winds following a strong NW wind event (Fig 3b), which is representative of the type iii) intrusion, according to [15].

### Models

We used the current, temperature and salinity fields computed by the RHOMA version of the model MARS3D on a horizontal grid extending from the Rhône River to Cap Sicié, with 30 vertical sigma layers, and a fine horizontal resolution of 200 m or 400 m, with corresponding time steps of 60 s or 30 s, respectively. This model, described in detail in [8,18], takes into account the forcing by the NW Mediterranean general circulation, on the basis of a nesting strategy with the large scale MARS3D-MENOR configuration (1.2 km resolution), by the atmospheric fluxes from the MM5 meteorological model (3 km resolution) and the average daily inputs of the Rhône River. We considered the 2 sets of results computed by the RHOMA model corresponding to the 2 selected study periods (see above), considered as representative of strong intrusions of the NC on the shelf associated with S-SE and E-SE wind conditions.

The Lagrangian trajectories of the fine suspended particles through the bays of Marseille were computed with the ICHTHYOP software, developed by IRD and PREVIMER and described in [19]. Ichthyop is an Individual-Based Model (IBM) with various sub-models including biological behaviors. Here, we used only the movement sub-model that simulates the following processes: horizontal and vertical advection, horizontal and vertical dispersion. In our study, horizontal and vertical advection were used in the movement equation but no horizontal or vertical dispersion was applied. Particles were considered as passive tracers. For time-stepping, a fourth order Runge–Kutta integration scheme was used with a constant time step of 30 s that respected the CFL criterion on the entire domain. In our study, the ICHTHYOP model was run taking into account the 3-hour resolution fields of Eulerian current velocities, temperature and salinity, previously computed by the MARS3D. In ICHTHYOP, these fields are interpolated in space to provide values at any individual (particle) location. They are also interpolated in time to feed the IBM time step (30 s) that addresses sub-grid scale processes. Simulations consist of tracking the locations and properties of the individuals (particles), typically during periods from a few hours to a few weeks. The output time step was 3 hours. Each computation took into account the same design for the initial source located at the WWTP outlet (Fig 1): a circular patch of 5x10^4^ particles with a diameter equal to a half mesh size (eg. 100 m or 200 m for the version of the model used), located at 1 m depth with a vertical thickness of 1 m. In addition, in each ICHTHYOP computation we considered the particles as passive elements in suspension without any biogeochemical transformation, with no buoyancy and no sinking velocity. All the Lagrangian trajectories were computed by ICHTHYOP as direct trajectories, with particles being transported from a single source, still located at the surface (1 m depth) at the WWTP, towards their respective targets within the bays. In addition, as the time of release strongly impacts the trajectories and final destination of particles, various tests were made on the time of the release for each event considered, and a choice was made to select the timing associated with trajectories that best transport the particles further up the bays. The source files of all the particle trajectories computed by ICHTHYOP in our study are available within the Supporting information S1 Dataset, with the two netcdf files S1A and S1B corresponding to the computations of the entire chosen periods of June 2008 and October 2011, respectively.

Finally, the number of suspended particles reaching each regional box shown in Fig 1, during each Lagrangian transport computation, is then counted and compared to the number of particles constituting the initial patch.

### Particulate organic matter

The ratios of the stable isotopes of carbon (^13^C/^12^C) and nitrogen (^15^N/^14^N) allow the identification of Particulate Organic Matter (POM) sources in coastal waters. Different literature data from the same season and area were used to identify the potential sources of the POM in suspension in the surface sea water at Somlit station (Fig 1). The stable isotope ratios of the marine phytoplankton (δ^13^C = -20.64+0.12 ‰, δ^15^N = 3.90+0.06 ‰) measured by [20], offshore, in the euphotic zone at the maximum of Chl*a* (at 90**–**100 m isobaths), may be associated with the marine phytoplankton transported by the NC. The continental river inputs of POM (δ^13^C = -26.32+0.89 ‰, δ^15^N = 5.19+1.56 ‰ and δ^13^C = -26.25+0.51 ‰, δ^15^N = 4.48+0.41 ‰, respectively) are mainly represented by various types of detritus, mainly of terrestrial plants (mainly C3 photosynthetic type) and freshwater phytoplankton [21,22]. Measurements at the surface in the WWTP plume confirmed that POM was composed of small particles from 2 to 6 mm with a concentration of 2.12x10^5^ particles^**.**^*μ*L^–1^, and most of them (94%) were detritus particles and bacteria [22]. These particles and probably bacteria used for water treatment at the WWTP induce a particularly low δ^15^N ratio (0.59+0.85 ‰) compared to the other POM sources [22]. The δ^13^C ratio (-25.5+0.62 ‰) in the POM from the WWTP plume is lower than that of the Somlit POM (δ^13^C = -23.59+1.37 ‰) [22]. The Somlit POM is a mixture from different sources of particles and generally the mean δ^15^N (2.18+1.39 ‰) is intermediary between marine phytoplankton, WWTP effluents and continental outflows POM.

These literature data regarding sources were compared with the stable isotope ratios of the Somlit POM on October 17 2011 [23]. As no rain event or continental outflow occurred in the previous days at Marseille, marine phytoplankton and WWTP POM were identified as the 2 main potential sources of suspended POM at Somlit on October 17 2011. Estimation of WWTP POM percentage in Somlit POM was calculated using the mixing equations (adapted from [24], where X was the ratio (δ^13^C or δ^15^N); the difference up to 100% was the percentage of marine phytoplankton:

$$P\%=\frac{\left(XSomlitPOM-XMarinePhytoplankton\right)}{\left(XCortiouPOM-XMarinePhytoplankton\right)}\times 100$$

where X is the ratio (δ^13^C or δ^15^N).

Thus, P% is the percentage of WWTP POM in the pool of suspended POM sampled at Somlit, while the difference up to 100% is the percentage of marine phytoplankton.

## Results

### Start of the particle intrusion

**Fig 4:**
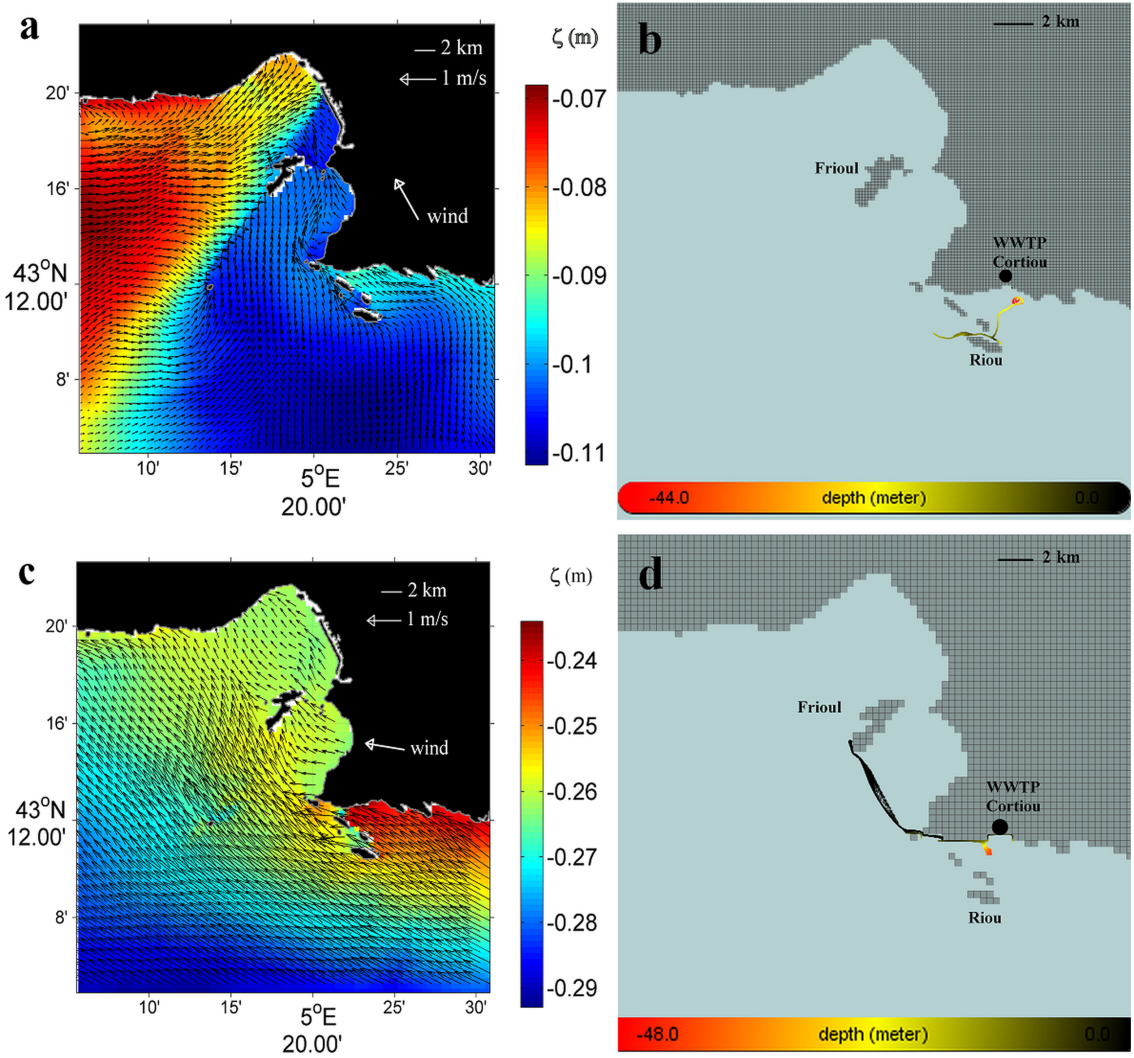
Modelling results of the starting intrusion (June 2008 and October 2011). (a) and (c): fields of surface current (arrows) and elevation (ζ) computed by MARS3D at the sea surface on June 17, 0:00, 2008, and October 15, 0:00, 2011, respectively. (b) and (d): trajectories of the passive particles computed by ICHTHYOP, relating to the same period as the corresponding current fields, on June 17, 6:00, 2008, and October 15, 0:00, 2011, respectively, over the 1–2 days following the particle release at the WWTP outlet; colors refer to the depths (m) reached by each particle *black* near the surface (< 10 m depth), *yellow* sub-surface (10–30 m depth) and *red* at depth (> 30 m depth).

Fig 4a presents the fields of surface current (arrows) and elevation (ζ) computed by MARS3D at the sea surface on June 17, 0:00, 2008. Fig 4b presents the positions of the passive particles computed by ICHTHYOP relating to the same period as the corresponding current field, on June, 17, 6:00, 2008, which corresponds to a time lapse of 54 hours after the particle release at the WWTP outlet on June 15, 0:00, 2008.

Fig 4c and 4d present the fields of surface current (arrows) and elevation (ζ) computed by MARS3D and the corresponding positions of the passive particles computed by ICHTHYOP on October 15, 0:00, 2011, respectively. This period corresponds to a time lapse of 18 hours after the particle release at the WWTP outlet on October 14, 6:00, 2011.

These results illustrate the first starting step of the process of the Lagrangian transport of passive particles released at the surface at the WWTP outlet, over the first 2 days after the particle release, during 2 different SE wind induced periods of intrusion of the NC on the shelf. Note that both cases considered, in June 2008 and in October 2011, illustrate specific modes of behavior of the particles, just after being released at the WWTP outlet, according to the crossshore (longshore) S-SE (E-SE) wind induced hydrodynamics along the southern coastline.

Fig 4a, in June, 2008, shows the surface circulation of the NC entering the bays of Marseille with current velocities ranging from 0.10 m.s^-1^ to 0.30 m.s^-1^ and rising sea surface elevations (ζ) along the southern coastline near the WWTP outlet, leading to an increased crossshore sea surface slope of about 2 cm by 4 km. This hydrodynamic situation occurred during a period characterized by a moderate S-SE 130°**-**170° winds < 7 m.s^-1^ hourly averaged speed, measured at the Frioul meteorological station.

Fig 4b confirms that the S-SE wind induced hydrodynamics in June 2008, characterized by onshore increasing sea surface elevations (ζ) and sea water accumulation near the WWTP outlet, promotes the rapid sinking of the released particles down to about 40 m depth under the effect of the local S-SE wind**-**induced downwelling. Then, Fig 4b shows the particles being transported about 4 km offshore as far as the vicinity of Riou island, where they can join the westward circulation of the NC at about 20 m depth. It should be noted that the sea water accumulation on the southern coastline and the resulting downwelling are reinforced during this period by the lower angles of incidence (less than 50°) of the S-SE wind induced surface currents with respect to the east-west orientation of the southern coastline near the WWTP outlet, that corresponds S-SE wind directions higher than 120° (Figs 3a and 4a).

Fig 4c, inversely, shows in October 2011 an intensive and westward surface circulation along the southern coastline near the WWTP outlet, with maximum current velocities of 0.70 m.s^-1^ computed at 1 m depth on October 15, 0:00, 2011 at a station chosen onshore close to the WWTP outlet (43.203°N / 5.362°E). This hydrodynamic situation occurred during a period characterized by a moderate E-SE 80°**-**110° winds < 5 m.s^-1^ hourly averaged speed, measured at the Frioul meteorological station.

Fig 4d shows an intense starting of the Lagrangian transport, but with particles remaining at the surface and being directly and quickly transported northwestwards from the WWTP outlet to the southern end of the Frioul archipelago, which can be reached in less than 1 day. It should be noted that this westward jet along the southern coastline is reinforced at this period by the higher angles of incidence (more than 50°) of the E-SE wind induced surface currents with respect to the east-west orientation of the coastline near the WWTP outlet, that corresponds, as seen above, to E-SE wind directions lower than 120° (Figs 3b and 4c).

### Development of the particle intrusion

**Fig 5:**
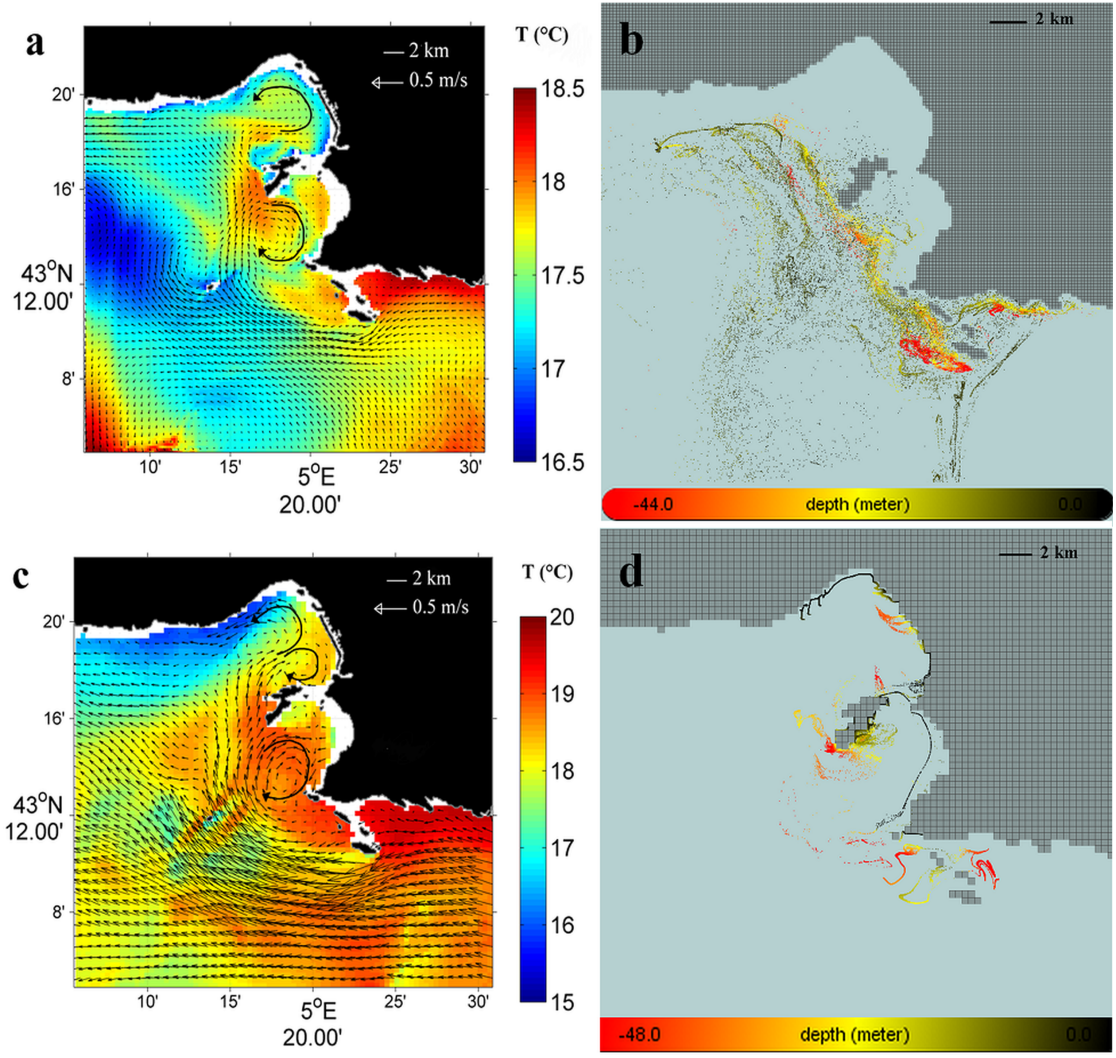
Modelling results of the developed intrusion (June 2008 and October 2011). (a) and (c): fields of current (arrows) and temperature (T°C) computed by MARS3D at 20 m depth on June 25, 9:00, 2008, and October 15, 18:00, 2011, respectively. (b) and (d): trajectories of the passive particles computed by ICHTHYOP, relating to the same period as the corresponding current fields, on June 22, 15:00, 2008, and October 15, 18:00, 2011, respectively, over the 2–7 days following the particle release at the WWTP outlet; colors refer to the depths (m) reached by each particle: *black* near the surface (< 10 m depth), *yellow* sub-surface (10–30 m depth) and *red* at depth (> 30 m depth).

Fig 5a presents the fields of current (arrows) and temperature (T°C) computed by MARS3D at 20 m depth on June 25, 9:00, 2008. Fig 5b presents the positions of the passive particles computed by ICHTHYOP relating to the same period as the corresponding current field, on June 22, 15:00, 2008, which corresponds to a time lapse of 7 days after the particle release at the WWTP outlet on June 15, 0:00, 2008.

Fig 5c and 5d present the fields of current (arrows) and temperature (T°C) computed at 20 m depth by MARS3D and the corresponding positions of the passive particles computed by ICHTHYOP on October 15, 18:00, 2011, respectively. This period corresponds to a time lapse of 36 hours after the particle release at the WWTP outlet on October 14, 6:00, 2011.

These results illustrate the second developing step of the process of the Lagrangian transport of passive particles sub**-**surface (about 20m depth) throughout the bays of Marseille, during SE wind induced periods of intrusion of the NC on the shelf. Note that both cases considered, in June 2008 and in October 2011, illustrate specific dynamics of particle transport that differ spatially and temporally.

Fig 5a clearly shows the intrusion of the NC sub-surface within the bays of Marseille, with northwesterly currents reaching, for example, a maximum computed velocity of 0.13 m.s^-1^ at 20 m depth in June 2008 at a chosen station in the southern bay at 43.20°N / 5.27°E. At this stage of development of the intrusion, the sub-surface currents are characterized by eddy circulations in the northern and southern bays, with an anticyclonic eddy in the southern bay, and 2 cyclonic and anticyclonic eddies in the more complex northern bay. In addition, the field of temperature (T°C) in Fig 5a shows the extension of warmer sea waters flowing westward and northwestward along the shoreline with a maximum crossshore temperature gradient reaching 1.5°C. This temperature gradient confirms the intrusion into the bays of Marseille of allochtonous water masses transported by the NC, which is characterized by Levantine waters, warmer than the waters of the NW Mediterranean continental shelf.

Fig 5b confirms the broad northwestward extension throughout the bays of Marseille of the fine particles released at the WWTP outlet, that within 1 week, in June 2008, and mostly sub-surface, reach the southern coastline of the Frioul archipelago (5 days) and the inner shorelines of the southern bay (8 days).

Fig 5c, in October 2011, shows the intensive intrusion of the NC within the bays of Marseille, with for example, a maximum current velocity of 0.49 m.s^-1^, computed at 20 m depth on October 15, 18:00, 2011 at the chosen station in the southern bay and located at 43.20°N / 5.27°E. At this stage of development of this rapid intrusion, the currents are clearly characterized by a well-shaped anticyclonic and cyclonic eddies in the southern and northern bays, respectively.

Fig 5d shows the further northward extension of the particles, but unlike in June 2008, the cloud of particles separates into 2 parts, one remaining near the surface (< 10 m depth) and the other sinking deeper in the water column to about 20 m depth, due to the local downwelling induced by the SE wind along the southeastern coastline of the Frioul archipelago. The particles remaining near the surface (< 10 m depth) are transported very rapidly, in less than 30 hours, to the limits of the northern and the southern bays along trajectories passing west and east of the Frioul archipelago, respectively. By contrast, the particles sinking in the downwelling slowly extend at sub-surface (about 20 m depth) along the eastern coastline of the Frioul archipelago only, where they can partly join the anticyclonic sub-surface circulation induced by the intrusion of the NC within the southern bay. In fact, the most striking result evidenced by this computation in October 2011, is to highlight the succession of 2 different modes of particle transport from their release at the WWTP outlet: i) the rapid E-SE wind induced westward transport at the surface of the whole patch along the southern coastline; ii) the extension of the transport towards the inner northern and southern bays as a result of the intrusion of the NC, but at different depths depending on whether the particles remain at shallower depths (< 10 m) or sink deeper owing to the downwelling at the southeastern coastline of the Frioul archipelago (about 20 m depth).

The ending of the process of particle transport by intrusion of the NC (not shown) is characterized by an extensive dissemination of particles at shallow depths (< 20 m) throughout the entire northern and southern bays, induced by internal residual circulations after the NC current is redirected to the west; in fact, the 2 intrusion events of the NC within the bays of Marseille considered in our study ceased on June 27, 12:00, 2008, and October 18, 2011, respectively.

### Time series of landing particle amounts

**Fig 6:**
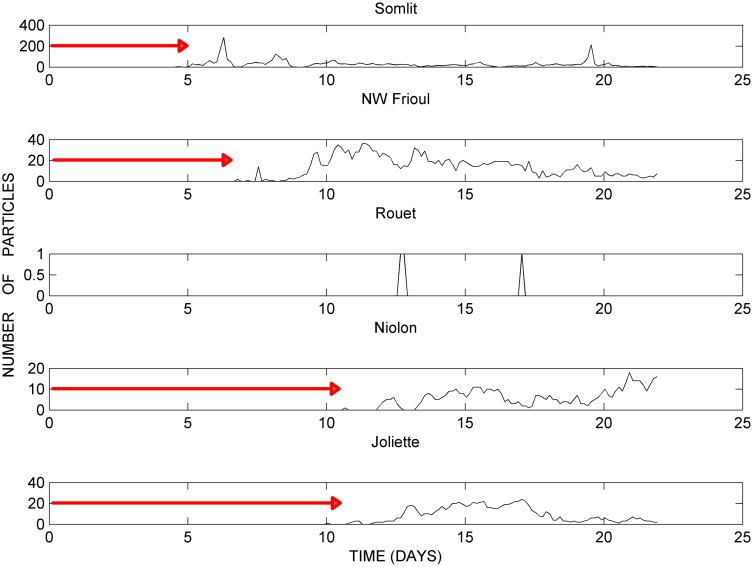
Time series of particle amounts reaching the western limit and northern bay. Computation from June 15, 0:00 to July 7, 0:00, 2008; areas impacted: Somlit, NW Frioul, Rouet, Niolon and Joliette; *red arrows* refer to the time lapse since the particle release.

**Fig 7:**
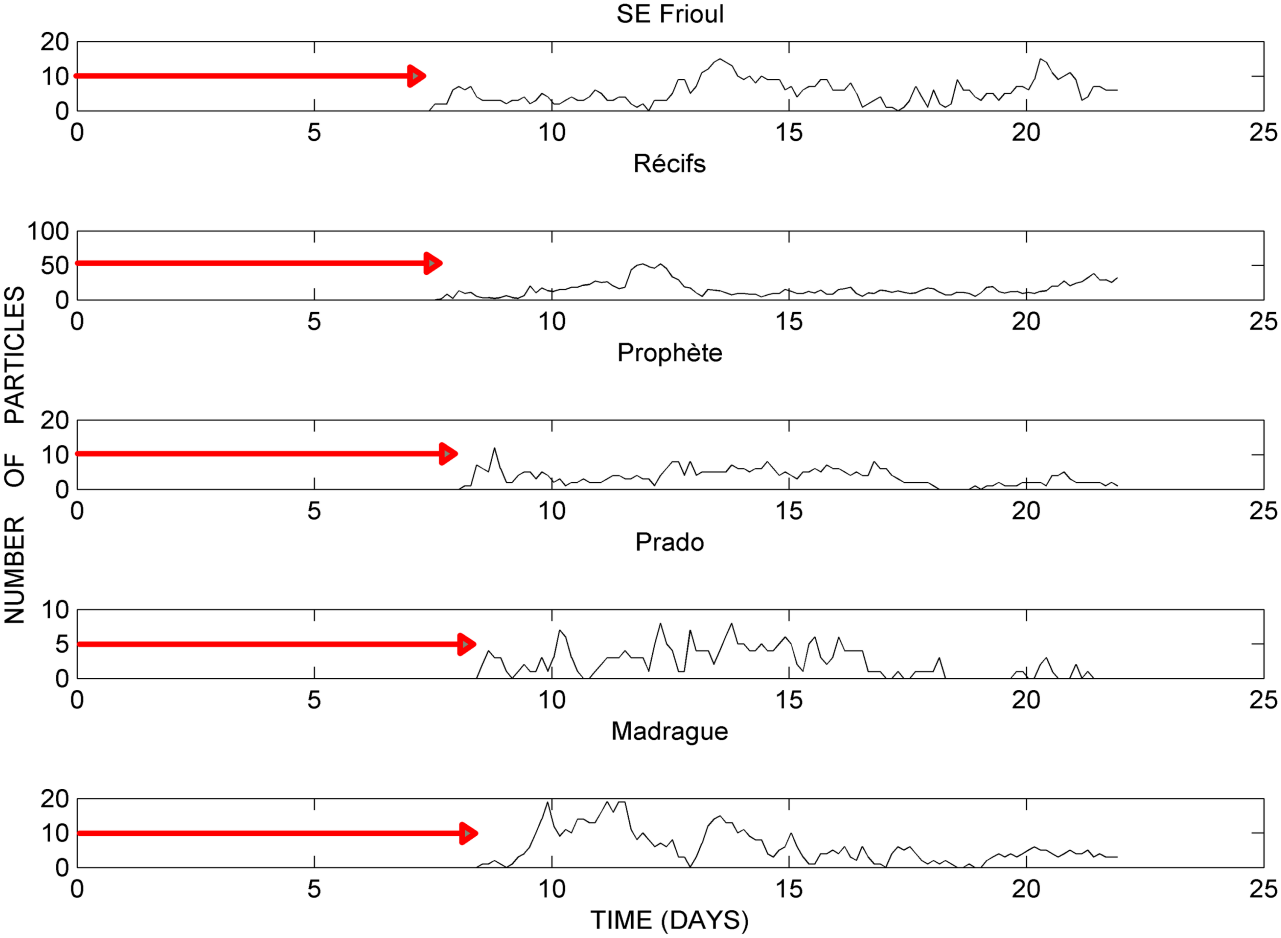
Time series of particle amounts reaching the southern bay. Computation from June 15, 0:00, to July 7, 0:00, 2008; areas impacted: SE Frioul, Récifs, Prophète, Prado and Madrague; *red arrows* refer to the time lapse since the particle release.

Figs 6 and 7 present, as an example, the time series of the amounts of particles that reach each of the 10 boxes considered around the bays of Marseille (Fig 1 and Table 1), only during the computation from June 15, 0:00, to July 7, 0:00, 2008, while the values computed in October, 2011 present quite similar shapes for each box considered. Stations are ranked in Figs 6 and 7 according to the order by which the particles reach them, and all the corresponding time lags of each trajectory since the release of the particles are mentioned in the Figures. In fact, Fig 6 refers to the 5 boxes featuring the western limit of the domain and the northern bay (Somlit, NW Frioul, Rouet, Niolon and Joliette), whereas Fig 7 refers to the 5 boxes representative of the shorelines located around the southern bay (SE Frioul, Récifs, Prophète, Prado and Madrague).

Results in Fig 6 highlight the progressive decrease of the landing particle amounts from the limit of the southern bay (Somlit) to the eastern limit of the northern bay (Joliette), following a path west of the Frioul archipelago. In fact, from the computation of June 2008, the maximum values of particle amounts decreased from 281 particles at Somlit to 36 particles at NW Frioul, 18 particles at Niolon and 24 particles at Joliette. Note that the landing particle amounts are quite similar at Niolon and Joliette, located on opposite sides of the northern bay, and that the landings at Rouet, located further westwards along the northern coastline, remain close to zero.

In addition, results in Fig 6 clearly show the increasing time lapse within which the particles reach the different locations around the bays, again along a gradient oriented from the limit of the southern bay (Somlit) to the eastern limit of the northern bay (Joliette). In fact, from the computation of June 2008, particles successively reach Somlit, NW Frioul and the northern coastline (Niolon and Joliette) within a time lapse of about 5, 7 and 10 days after their release at the WWTP outlet, respectively.

Results in Fig 7 illustrate the fact that along the shorelines of the southern bay, all the boxes are reached by particles at the same time, within 7 to 8 days after their release at the WWTP outlet, with a slight increase in the time lapse for the boxes located along the eastern shoreline (Prophète, Prado and Madrague) compared to western shoreline (SE Frioul) and the central area (Récifs).

In addition, results highlight that the central box corresponding to the artificial reefs (Récifs) is mostly impacted by particle landings shaped as 2 peaks of 50 and 40 particles, occurring within time lapses of 12 and 22 days after their release, respectively. In contrast, the other boxes representative of the shorelines around the southern bay are characterized by weak but regular particle landings, less than 20 particles at Madrague, decreasing to less than 10 particles at Prophète and Prado.

### Cumulative amounts of landing particles

**Fig 8:**
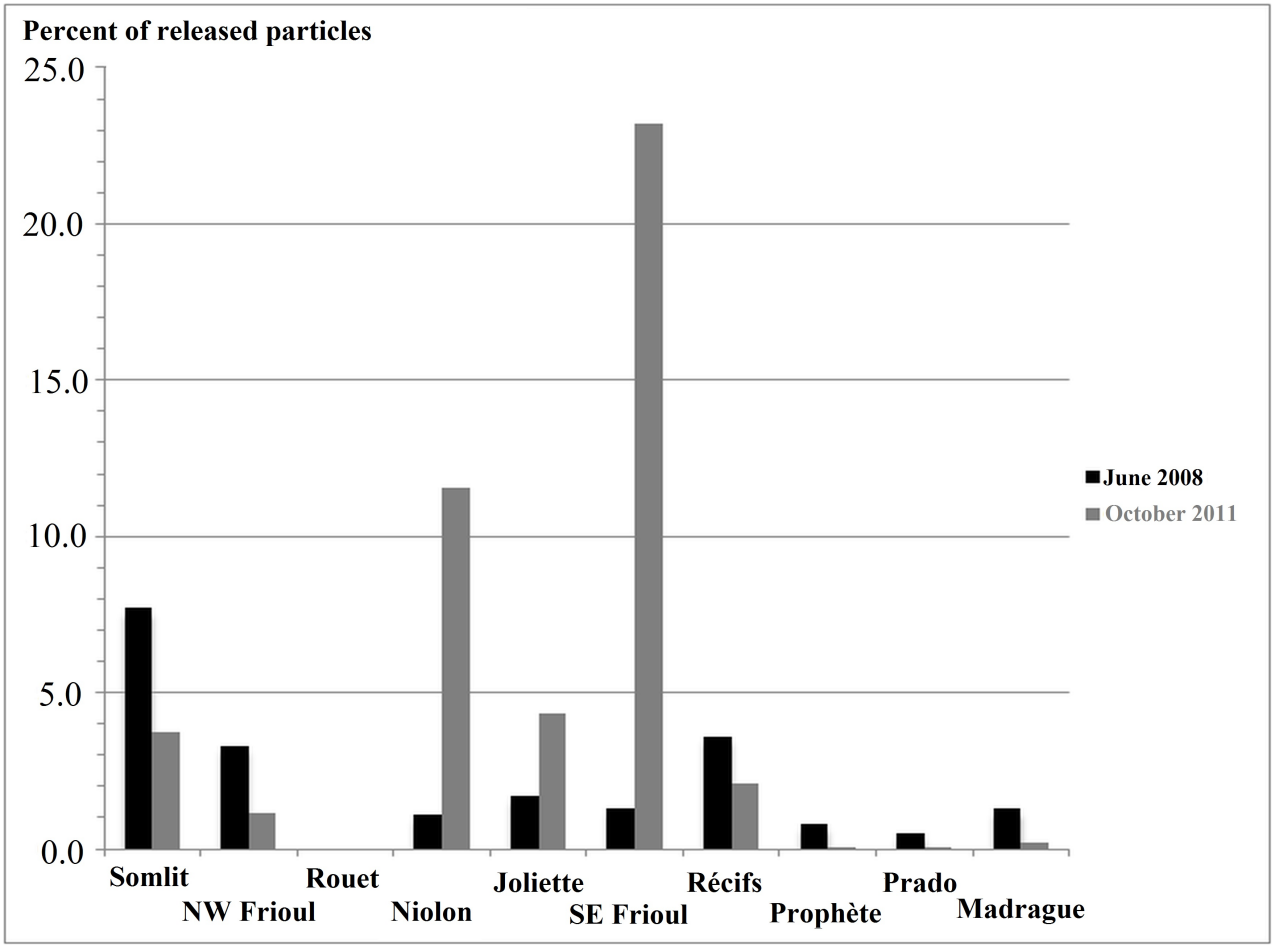
Spatial distribution of the WWTP initial patch of particles. The amount of particles reaching each coastal site is expressed in terms of fractions (%) of the initial patch of 5.10^4^ particles, during the computations from June 15, 0:00 to July 7, 0:00, 2008 and from October 14, 6:00 to October 20, 0:00, 2011.

Fig 8 presents for each box considered, the proportions of the cumulative amounts of landing particles expressed in terms of fractions (%) of the initial patch of 5.10^4^ particles, during the computations from June 15, 0:00, to July 7, 0:00, 2008 and from October 14, 6:00, to October 20, 0:00, 2011.

Results in Fig 8 confirm different modes of particle dispatching within the northern and southern bays, comparing the intrusions of the NC in June 2008, and October 2011.

Results for June 2008 in the western limit and the northern bay show the progressively decreasing impact of the WWTP effluent following a northeastward gradient, from a value of 7.7% for the initial patch at the limit of the southern bay (Somlit) to values of 3.3% along the western coastline of the Frioul archipelago (NW Frioul), and about 1% and 1.7% on the opposite sides of the northern bay, at Niolon and Joliette, respectively. Note than the results close to zero at the box Rouet confirm the existence of a spatial limit of the impact of the WWTP, at an undefined location between Niolon and Rouet on the northern coastline.

Results for June 2008 in the southern bay show a dominant impact of the WWTP effluent in the central area corresponding to the artificial reefs (Récifs), with the maximum value of 3.6% for the initial patch. Moreover, results present a northward decreasing gradient of the WWTP impact along the 2 opposite shorelines of the southern bay, with values ranging from about 1.3% of the initial patch at SE Frioul and Madrague, and decreasing to less than 1% of the initial patch at Prophète and Prado.

Results for October 2011 in the western limit and the northern bay, unlike results shown for June 2008, do not present any spatial gradient of the WWTP impact, but show moderate values of proportion of the initial patch at Somlit (3,7%) and NW Frioul (1%), and this time, peak values up to 11.5% 4.3% along the 2 opposite sides of the northern bay, at Niolon and Joliette, respectively.

Results for October 2011 in the southern bay, unlike results for June 2008, evidence a mode of distribution of the particles characterized by an eastwards decreasing gradient of the impact levels along the shorelines of the southern bay, from the peak value of 23.2% of the initial patch at SE Frioul to values of the same magnitude as in June 2008 at Récifs (2.1%), and then values becoming close to zero at the eastern stations (Prophète, Prado and Madrague).

Finally, it is worth noting that our computations highlight the variability of the amounts of particles discharged at the WWTP at Cortiou that reach the inner shorelines of the bays of Marseille, with total proportions, taking all boxes added together, ranging from a total of 21.2% of the initial patch in June 2008, up to 46.3% of the initial patch in October 2011.

### Particulate organic matter

**Fig 9:**
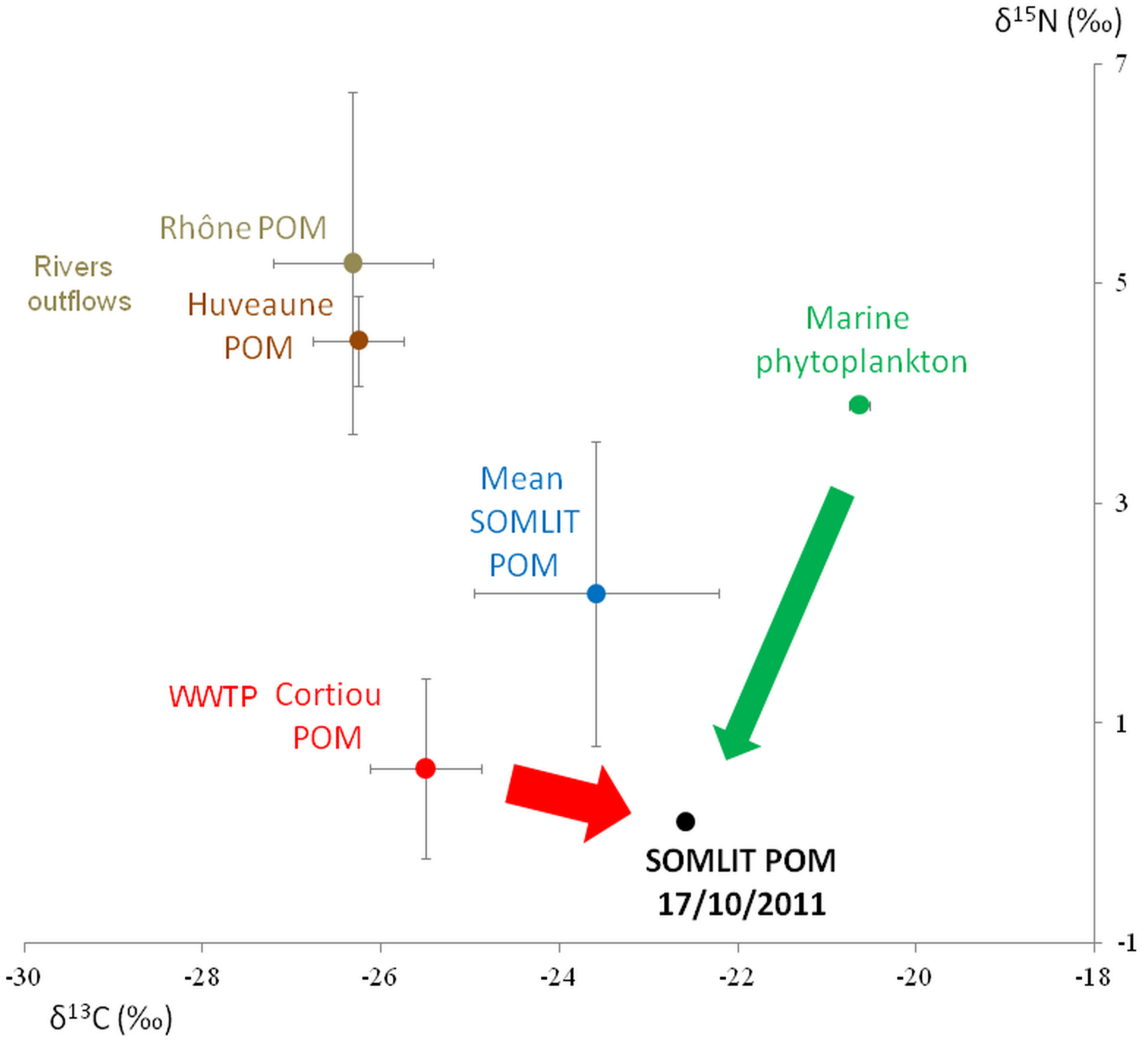
Stable isotope ratios of the surface seawater POM. Sampling at Somlit on October 17, 2011; comparison with ratios of the main sources of POM identified in the bays of Marseille; the 2 main sources of POM are indicated by arrows.

Fig 9 presents the stable isotope ratios of the main sources of POM in the bays of Marseille from the literature, and from the surface seawater POM sampled at Somlit (Fig 1), on October 17, 2011. The 2 identified main sources of suspended POM sampled at Somlit during that period were marine phytoplankton and WWTP effluent POM. The δ^13^C stable isotope ratio of the POM at Somlit on October 17, 2011, was higher than the mean value of POM at Somlit estimated in [22], and lower than the mean value of marine phytoplankton estimated in [20]. The δ^15^N stable isotope ratio of the POM at Somlit on October 17, 2011, was even lower than the mean δ^15^N of WWTP effluent POM.

The mixing equations applied to the sampled POM at Somlit using δ^13^C estimated the proportion of the WWTP effluent at 40%, while when using δ^15^N it was estimated at 100%. Similarly, the proportion of the marine phytoplankton was estimated at 60% when using δ^13^C ratios.

## Discussion

Our approach remains schematic but results reveal, by way of example, that the potential impact of an urban effluent such as that of the WWTP outlet at Cortiou, near Marseille, may represent a major concern in terms of health risk for most of the coastal activities of such a large city. Our study takes into account the specific hydrodynamic context which is the only one allowing the transport of particles from the WWTP effluent towards the inner bays of Marseille: a period of intrusion of the NC on the continental shelf associated with a SE wind regime.

### Characterization of the NC intrusions

We selected our 2 events of intrusion of the NC within the bays of Marseille based on the computed velocity fields (RHOMA model), which exhibited a clear on**-**shelf velocity component at the entrance of the southern bay. The 2 intrusion events had an impact over the entire water column with discernible onshore velocity components computed from 20 m depth down to about 80 m [11].

Authors in [11] used the modeled meridian velocities to compute an average on**-**shelf (northward) flow of about 0.45 Sv [1 Sverdrup (Sv) = 10^6^ m^3**.**^s^-1^] across a line at 43.07° N from October 15 to 18, 2011, with short**-**term peak flows reaching about 0.65 Sv. Using the same method, and in order to compare our results with those described in [11], we computed that the intrusion flow can amount to an average value of about 0.30 Sv across a line at 43.07° N from June 15 to 28, 2008, with short**-**term peak values reaching about 0.38 Sv. In addition, these 2 events presented different intensities and directions of velocity field. At the station located close to the southern coastline near the WWTP outlet (43.203°N / 5.362°E), the current velocity was greater in October 2011, with maximum values of 0.49 m.s^-1^ at 20 m depth, whereas maximum current velocities were 0.13 m.s^-1^ at 20 m depth in June 2008.

These results confirm that the intensity of NC intrusions are not directly driven by SE winds conditions as shown by the authors of [15]. In fact, the intensity of SE winds during the period of June 2008 was greater than in October 2011. Inversely, the intensity of the NC intrusion in June 2008 was weaker (in terms of currents and northward flow) than the intensity of the NC intrusion in October 2011. Our results confirm that in October 2011, the intrusion of the NC was triggered by a strong NW wind event until October 14, 2011, and was then reinforced by the SE wind that followed as described in [11]. This type iii) of wind combination accelerates the intrusive current as described in [15], giving rise to stronger current intensity.

On the contrary, in June 2008, the intrusion of the NC is related to a downwelling induced by S-SE winds as described above and in the literature. Authors in [25] suggested that the Ekman transport associated with SE winds induces a downwelling, which generates a westward coastal current that transports the NC waters onto the shelf. This hypothesis was confirmed by authors in [15] in the case of an intrusion induced by SE winds, associated with sea water accumulation along the southern coastline that drives a longshore shelf-intruding current, occurring independently of the stratification and concomitantly with the wind forcing.

### Spatial variability of particle connectivity

Results highlight that the northern and the southern bays of Marseille are not impacted in the same way, revealing that the connections of particles from the WWTP effluent result from the complex interaction between SE wind induced current fields and the circulation induced by intrusions of the NC on the shelf.

Firstly, as seen in Figs 6 and 8, the hydrodynamics induced by a NC intrusion and a E-SE wind (as seen in October, 2011), promotes rapid surface, and then near-surface (< 10 m depth), particle connections that are more efficient at accumulating particles along the southeastern coastline of the Frioul archipelago (SE Frioul) than entering the southern bay (Prophète, Prado, Madrague). In addition, this type of near-surface circulation is particularly favorable for transporting more particles to the farthest shorelines of the northern bay (Niolon, Joliette).

Inversely, as seen in Figs 7 and 8, the hydrodynamics induced by a NC intrusion and a S-SE wind (as seen in June, 2008), promotes slower particle connections sub-surface (about 20 m depth) that are more efficient at entering the southern bay and dispatching particles all along the inner shorelines (Madrague, Récifs, Prophète, Prado). In addition, this type of sub-surface circulation following trajectories located a little further south than those induced by E-SE winds, is particularly favorable for transporting more particles to areas located south and west of the Frioul archipelago (Somlit, NW Frioul), with the same amount of particles connecting NW Frioul and Récifs, located on each side of the Frioul archipelago.

Secondly, our results in Fig 8 show that stations that are geographically distant from one another may be connected by the same number of particles, and much more than stations that are close to them. For example in October 2011, the remote stations Somlit and Joliette connect in the same way and much more than NW Frioul; in the same way in June 2008, the remote stations Madrague and Joliette connect in the same way and much more than Prophète.

In addition, the results in Figs 6 and 8 show a kind of ‘boundary’ of connectivity between Niolon and Rouet, which conversely are stations geographically close to one another along the northern coastline; in fact, Niolon is highly impacted by the effluent, while Rouet remains without any particle connection according to both computations in June 2008 and October 2011. Note that in both computations in June 2008 and October 2011, the eastern limit of the northern bay (Joliette) is strictly impacted from the west, according to the circulation induced by the NC intrusion on the shelf, and without any connection from the central southern bay (Récifs), despite the geographical proximity of these 2 locations.

Therefore, all these results should be displayed in the form of a conceptual diagram to present 2 modes of particle connectivity from the WWTP outlet to the inner shorelines of the bays, in both cases under periods of intrusion of the NC on the shelf, but depending on the S-SE or E-SE wind direction.

### Conceptual diagram of particle connectivity

**Fig 10:**
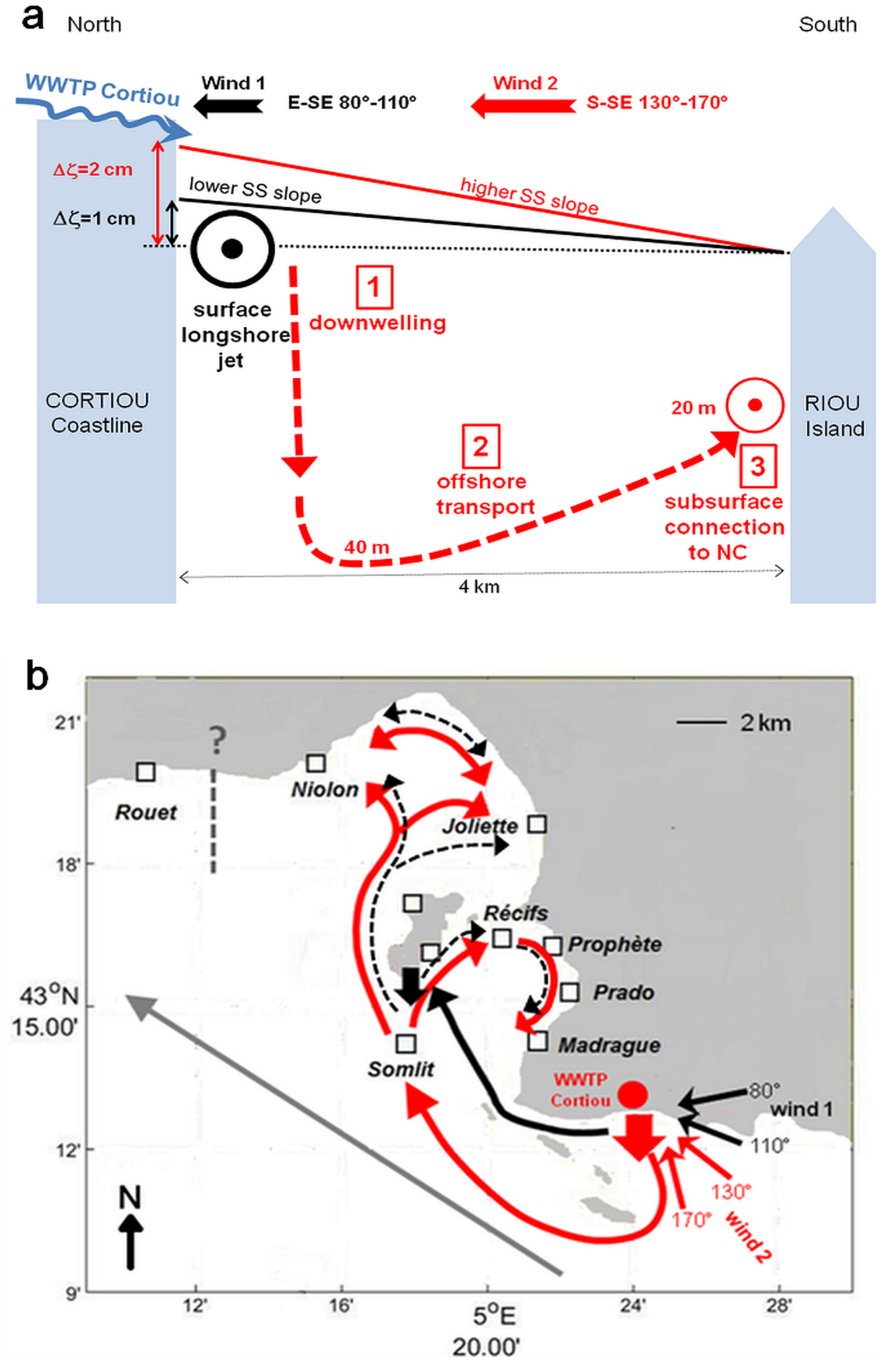
Conceptual diagram of connectivity from the WWTP outlet. (a): diagram showing the start of particle transport in the vicinity of the Cortiou WWTP outlet, following 2 paths according to E-SE (wind 1) and S-SE (wind 2) wind conditions. (b): comparative diagram of particle connectivity from the Cortiou WWTP outlet with and without intrusion of the NC on the shelf and associated with E-SE and S-SE winds; *grey path*: particle transport at the surface induced by SE winds without NC intrusion; *solid black path*: particle transport at the surface induced by NC intrusion and E-SE wind; *dotted black path*: particle transport near the surface (< 10 m depth) induced by NC intrusion and E-SE wind; *red path*: particle transport sub-surface (about 20 m depth) induced by NC intrusion and S-SE wind; *thick black arrow* refers to the downwelling at the southeastern coastline of the Frioul archipelago; *thick red arrow* refers to the downwelling front of the WWTP outlet; *grey dotted line* refers to the presumed northwestern limit of the WWTP impact zone.

Fig 10a presents a conceptual diagram showing the start of the particle transport in the vicinity of the Cortiou WWTP outlet, following 2 types of path according to E-SE or S-SE winds. This diagram offers a clearer explanation of the S-SE wind induced downwelling process in front of the WWTP outlet and the resulting sub-surface particle transport, versus the E-SE wind induced longshore jet and the resulting westward particle transport at the surface.

On the one hand, an E-SE 80°-110° wind regime (Fig 10a, wind 1 and *black path*), as studied in October 2011, which is characterized by higher angles of incidence (60°-90°) relative to the orientation of the southern coastline, locally induces a coastal circulation characterized by northwesterly currents (Fig 4c), a lower crossshore sea surface slope of about 1 cm by 4 km, with lower water accumulation onshore and an intense longshore jet that rapidly transports the particles westwards at the surface and close to the southern coastline, from the WWTP outlet straight to the south end of the Frioul archipelago, which is reached in 18 hours (Fig 4d).

On the other hand, a S-SE 130°-170° wind regime (Fig 10a, wind 2 and *red path*), as studied in June 2008, which is characterized by lower angles of incidence (0°-40°) relative to the orientation of the southern coastline, locally induces a coastal circulation characterized by northward currents (Fig 4a), a higher crossshore sea surface slope of about 2 cm by 4 km, with higher water accumulation onshore associated with a downwelling circulation that causes the particles from the WWTP outlet to sink down to 40 m depth. Then, particles are transported offshore to the north of Riou island in 2 days, where they may join at about 20 m depth the sub-surface westward circulation induced by the intrusion of the NC on the shelf (Fig 4b).

Fig 10b presents a comparative diagram showing the different paths of the particle connectivity from the Cortiou WWTP outlet with and without intrusion of the NC on the continental shelf and still associated with E-SE and S-SE winds.

Firstly, under a SE wind regime but in the absence of intrusion of the NC on the continental shelf, the particles are directly and rapidly transported at the surface to the north-west (Fig 10b, *grey path*), along trajectories passing south of the Frioul archipelago without entering the inner bays [16].

Secondly, in periods of intrusion of the NC on the continental shelf, from the moment the particles reach the southern end of the Frioul archipelago, and whatever their depth, they continue to be transported northward as a result of the intrusion of the NC within the bays (Fig 5a and 5c), but at different depths depending on the starting mode that they followed according to the prevailing wind regime at the moment of their release at the WWTP outlet (Fig 10a).

In a period of intrusion of the NC associated with a E-SE 80°-110° wind regime, as studied in October 2011, particles reaching the Frioul archipelago at the surface (Fig 10b, wind 1 *solid black path*), sink slightly deeper (< 10 m depth) under the effect of the wind induced downwelling that prevails along the southeastern coastline of the Frioul archipelago (*thick black arrow*). Then these particles continue to move rapidly northwards, passing west (east) of the Frioul archipelago to the extreme (inner) shorelines of the northern (southern) bay, which are reached in less than 30 hours (Figs 5d and 10b *dotted black paths*).

By contrast, in a period of intrusion of the NC associated with a S-SE 130°-170° wind regime, as studied in June 2008, particles reaching the Frioul archipelago sub-surface (Fig 10b, wind 2 *red path*) continue to move northwards at the same depth (about 20 m depth) on both sides of the Frioul archipelago, either to disperse throughout the southern bay according to the local anticyclonic circulation, or to reach the distant shorelines of the northern bay within approximately 8 days (Figs 5b and 10b *red paths*).

Note that a fraction of the patch of particles that reach the southeastern end of the Frioul archipelago at the surface (Fig 10b wind 1 *solid black path*), sink deeper in the water column to about 20m depth (*thick black arrow*) that leads them to join the sub-surface (about 20 m depth) northward circulation of the intrusion of the NC, which enters deeper into the southern bay (Figs 5b and 10b *red path*).

In addition, as shown in Figs 6, 7 and 8, our results show that Rouet is not impacted by the particle connections, which suggests locating the spatial limit of the impact zone of the WWTP effluent somewhere along the northern coastline between Niolon and Rouet (*grey dotted line*).

Although these 2 proposed modes of particle transport from the WWTP outlet to the bays of Marseille present a similar pattern, since they are both controlled by the intrusion of the NC within the bays, they differ essentially in the depth at which the particles are transported, and the resulting speed of their transport. In addition, it is interesting to note that our results identify 2 specific locations of downwelling, where particles from the WWTP outlet sink to sub**-**surface depths, according to the angle of incidence of the SE winds, and resulting surface currents, with respect to the orientation of the southern coastline at the site of the WWTP outlet. In summary, an E-SE wind with higher incidence (direction < 120°) will induce delayed particle sinking and shallower and faster connectivity, especially effective in reaching the distant shorelines of the northern bay, while a S-SE wind with lower incidence (direction > 120°) will induce immediate particle sinking and deeper and slower connectivity, especially effective in reaching the inner shorelines of the southern bay.

### Confirmation of the particle origins

On the basis of the measurements of stable isotopes, our results confirmed that the POM at Somlit is partially constituted of particles transported from the WWTP effluent. As suggested by the δ^13^C stable isotope ratio, the sea surface POM, which arrived at Somlit on October 17, 2011, was probably constituted by a mixture of marine phytoplankton transported by the NC and detritus particles from the WWTP effluent. Moreover, the δ^15^N stable isotope ratio of POM at Somlit was particularly influenced by the POM from the WWTP plume. These particles are consumed by the zooplankton and integrate the pelagic food web particularly during the cold season (fall and winter) [22]. These particles may also be consumed by benthic filter feeder organisms after their release at the WWTP outlet [26]. A mixture of marine phytoplankton and a large part of the POM from the WWTP effluent may be consumed by the filter feeder organisms in the artificial reefs (Récifs), even if the main source of this food web suggested by the authors is the nanophytoplankton [27]. In fact, according to these authors, the estimated value of the basis of the food web (δ^13^C = -25.23±1.16 ‰, δ^15^N = 1.77±0.25 ‰) is rather close to values of the POM from the WWTP effluent at Cortiou (δ^13^C = -25.5±0.62 ‰, δ^15^N = 0.59±0.85 ‰). The results of the present paper, relating to intrusions of the NC on the shelf and the high resulting impact of the particles from the WWTP effluent in the artificial reefs zone (Récifs), support this hypothesis.

Interestingly, hydrodynamic circulation and stable isotope ratios could be used at the same time to better identify the respective contributions of the different seawater sources of POM. For example, in October 2001, at Somlit: 40% and 60% of the sampled POM may be attributed to particles from the WWTP effluent and marine phytoplankton, respectively. Thus, Lagrangian trajectories computed in this study confirm that a significant part of the particles from the WWTP effluent are effectively connected to Somlit (3–7%), and furthermore the study published by [11] showed that the NC intrusion of October 17, 2011, induced an intrusion on the shelf of the warmer and mostly oligotrophic water from the NC, characterized at Somlit by its signature in terms of offshore marine phytoplankton.

### Concerns about human activities

From a health point of view, Niolon is a diving and bathing site that is potentially at risk of being contaminated by the particles from the WWTP outlet. Finally, it is crucial that Rouet, which is the site of a Protected Marine Area, is almost never impacted by the potentially polluted particles. Joliette is a commercial harbour that is more likely to be polluted by vessel traffic than by particles from the WWTP effluent.

In the southern bay, it should be noted that the sites the most impacted by the WWTP effluent are SE Frioul (especially in October, 2011) and Récifs, each characterized by vulnerable human activities such as fish farming (SE Frioul) and a zone of artificial reefs for production (Récifs). In addition, we highlighted that the sites Récifs (artificial reefs), and also Somlit (water quality monitoring station), both located in the middle of the southern bay, were highly impacted by particles from the WWTP effluent, especially in June 2008. This result reveals that the particles from the WWTP can potentially be integrated into the pelagic and the benthic food webs within the southern bay, where professional and recreational fishing is highly developed. Finally, bathing areas such as Prophète, Prado and Madrague, located at the southeastern limit of the anticyclonic sub-surface circulation induced by the intrusion of the NC within the southern bay, remain weakly impacted by particles from the WWTP effluent.

## Conclusion

The mechanism we propose in the present study for the dispersal of fine particles from the WWTP effluent at Cortiou throughout the bays of Marseille involves surface, shallow (< 10 m depth) and sub-surface (about 20 m depth) modes of particle transport, induced by intrusions of the NC on the continental shelf, associated with SE wind regimes. These modelling results are quite new and unexpected. In fact, the present study is of interest because the importance of the sub**-**surface circulation is too often under-estimated compared to the surface circulation, more intuitively considered, but very different from the deeper circulation and, in our case, ineffective in connecting sites located in the inner bays of Marseille. Our study highlights that the transport of particles from the WWTP effluent towards the inner shorelines of the bays of Marseille is not negligible. For each studied event, if we take together all the amounts of particles having connected the 10 vulnerable sites chosen around the northern and southern bays (Figs 6, 7 and 8), we obtain the relatively high values of 21.2% and 46.3% in June 2008 and October 2011, respectively. Moreover, the observations at Julio station (Fig 1) reveal that the occurrence frequency of the intrusions of the NC on the continental shelf is not so uncommon. In fact, the 3 series of measurements performed at Julio and covering 23 months between February 2012 and April 2015, show an average of 1 to 2 intrusions of the NC per month (A. Petrenko, pers. comm.). The intrusions of the NC on the shelf at sub-surface depths therefore represent a significant health risk in relation to coastal water quality along the shorelines of both the northern and southern bays of Marseille, and seeking greater knowledge in terms of their intensity, frequency and dynamics are ongoing research topics. At present, the analysis of the observations available at Julio station (2012–2015) is being processed in relation to the analysis of wind regimes and modeled current fields (A. Petrenko, pers. comm.). More generally, it is clear from our recent experience that enhanced monitoring of the sub-surface circulation, often under-estimated, with bottom-moored ADCP stations, as well as periodic analyses of the POM at sea surface with the stable isotope method, might play a major role in the detection and prediction of health risk episodes in a large number of highly urbanized coastal areas.

## Supporting information

S1 Dataset Source files of computed particle trajectories (ZIP file). S1A and S1B (netcdf) correspond to the ICHTHYOP computations of June 2008 and October 2011, respectively.
(ZIP)
